# Dietary BCAA Intake Is Associated with Demographic, Socioeconomic and Lifestyle Factors in Residents of São Paulo, Brazil

**DOI:** 10.3390/nu9050449

**Published:** 2017-05-02

**Authors:** Ana Carolina Pallottini, Cristiane Hermes Sales, Diva Aliete dos Santos Vieira, Dirce Maria Marchioni, Regina Mara Fisberg

**Affiliations:** Department of Nutrition, School of Public Health, University of Sao Paulo, Av. Dr. Arnaldo, 715, Cerqueira César, São Paulo CEP 01246-904, Brazil; anapallottini@hotmail.com (A.C.P.); cristianehermes@yahoo.com.br (C.H.S.); diva.nutricao@gmail.com (D.A.d.S.V.); marchioni@usp.br (D.M.M.)

**Keywords:** diet, epidemiology survey, nutritional assessment, protein

## Abstract

Background: Identifying which risk groups have a higher intake of branched chain amino acids (BCAA) is important for the planning of public policies. This study was undertaken to investigate BCAA consumption, the foods contributing to that consumption and their association with demographic, socioeconomic and lifestyle factors. Methods: Data from the Health Survey of São Paulo, a cross-sectional population-based survey (*n* = 1662; age range 12–97 years), were used. Dietary intake was measured using 24-h dietary recalls. Baseline characteristics were collected. Associations between BCAA intake and demographic, socioeconomic and lifestyle factors were determined using linear regression. Results: Total BCAA intake was 217.14 mg/kg·day (Leu: 97.16 mg/kg·day; Ile: 56.44 mg/kg·day; Val: 63.54 mg/kg·day). BCAA intake was negatively associated with female sex in adolescents and adult groups, with no white race in adolescents, and with former smoker status in adults. Conversely, BCAA was positively associated with household per capita income in adolescents and adults. No associations were observed in the older adults group. Main food contributors to BCAA were unprocessed red meat, unprocessed poultry, bread and toast, beans and rice. Conclusions: Adolescents and adults were the most vulnerable to having their BCCA intake influenced by demographic, socioeconomic and lifestyle factors.

## 1. Introduction

The branched-chain amino acids (BCAA) leucine (Leu), isoleucine (Ile) and valine (Val) are an important class of essential amino acids. These amino acids play an important role in the human body for protein synthesis, production of energy and synthesis of many neurotransmitters [1,2]. Moreover, the BCAA contribute to dietary protein intake [3], with animal protein being the main source

Several studies have shown that elevated levels of BCAA in blood, including Leu, Ile and Val, may be related to the risk of developing type 2 diabetes, insulin resistance and cardiovascular diseases [4,5,6,7]. These noncommunicable diseases (NCD) are the major causes of death worldwide. The World Health Organization has projected an increase in mortality from NCD from 38 million in 2012 to 52 million in 2030 [8].

Studies evaluating the dietary intake of BCAA are scarce. Moreover, despite the literature demonstrating the importance of identifying the relationship between dietary intake and socioeconomic status for advances of public health [9], there is a gap of knowledge regarding the association between macronutrient and socioeconomic status [10].

To our knowledge, there have been no population-based studies that examined the association of dietary BCAA intake with demographic, socioeconomic and lifestyle factors in Brazil. Therefore, the aim of this study was to investigate BCAA consumption and their association with demographic, socioeconomic and lifestyle factors, and to identify the main foods contributing to BCAA intake.

## 2. Materials and Methods 

This study was approved by the Ethics Committee of the Public Health School of the University of São Paulo (Certificate of Presentation for Ethical Appreciation—CAAE # 26800414.1.0000.5421). All participants provided written informed consent before all procedures.

### 2.1. Study Design

Data were drawn from the 2008 Health Survey of São Paulo (Inquérito de Saúde de São Paulo, ISA-Capital 2008). This is a cross-sectional population-based survey that used a complex, stratified, multistage probability sample of individuals living in permanent households from urban areas of Sao Paulo, in southeastern Brazil. In this survey, health, nutritional information and lifestyle conditions were collected.

The sample was obtained in two stages. As the primary sampling units, 70 census tracts were randomly selected from all urban census tracts in the city of São Paulo. In the second stage, 16,607 households were randomly selected within these census tracts and stratified. Eight domains were fixed according to age and sex: infants (<one year; both genders); children (1–11 years; both genders); male adolescents (12–19 years); female adolescents (12–19 years); male adults (20–59 years); female adults (20–59 years); male older adults (60 years or more) and female older adults (60 years or more). To preserve the representativeness of each domain and to correct differences in the relative participation of the age groups in the population of the city of Sao Paulo, different sampling fractions were applied. The sample size was calculated to estimate proportions of 0.5 with a sample error of 0.07 at a 5% significance level and design effect of 1.5.

This ISA-Capital study included 2691 individuals aged 12 years and older for both sexes (605 adolescents, 1162 adults and 924 older adults). In the present study, we included only data obtained from adolescents (*n* = 560), adults (*n* = 585) and older adults (*n* = 517) of both sexes, who answered a structured questionnaire and at least one 24-h dietary recall (24HR). The survey response rate was 77%. The findings presented here are based on the complete case analysis.

### 2.2. Data Collecting and Processing

Characteristics such as weight, height, physical activity level, sex, age, the household head’s level of education, household per capita income, self-reported race, alcohol consumption and smoking status were collected by trained interviewers using a structured questionnaire formulated for this study.

Age was calculated by the difference between the interview date and the date of birth. Self-reported race was classified as *white* and *non-white* (black, brown, yellow and indigenous). The household head’s level of education was measured in years of study. Household per capita income was calculated by adding the monetary income reported by all family members and dividing by the number of family members (1 minimum wage = US$260.00; R$415.00). The smoking status was obtained from questions about current or former smoking habits and the number of cigarettes smoked per day were classified as *never*, *current* and *former smoker status*. The intake of alcoholic beverages was obtained by asking questions about the amount, frequency and preference of the consumption of alcohol, and the individuals were classified as *consumer* or *non consumer* of alcoholic beverages [11].

Level of physical activity was collected by the long version of the International Physical Activity Questionnaire [12]. Leisure time physical activity was classified as *insufficiently active* or *sufficiently active* (*sufficiently active* was defined as physical activity practiced at least 30 min daily, five days per week, at a moderate intensity or at least 20 min daily, three days per week, at a vigorous intensity).

Body mass index (BMI) was calculated using the Quetelet equation (BMI = weight (kg)/height (m^2^)), from self-reported weight and height and classified according to the cut-off for adolescents (underweight, BMI < 3rd percentile; healthy weight, BMI > 3rd percentile and <85th percentile; overweight, BMI > 85th percentile and <97th percentile; obese, BMI > 97th percentile) [13], adults (BMI < 18.5 kg/m^2^; healthy weight, BMI 18.5–24.9 kg/m^2^; overweight, BMI 25.0–29.9 kg/m^2^; obese, BMI ≥ 30 kg/m^2^) [14] and older adults (underweight, BMI < 23 kg/m^2^; healthy weight, BMI 23.0–27.9 kg/m^2^; overweight, BMI 28–29.9 kg/m^2^; obese, BMI ≥ 30 kg/m^2^) [15]. This information was validated by Carvalho et al. [16] in a previous study with the same population, that found high sensitivity (>91%) and specificity (>83%) in all ages and sex groups.

### 2.3. Assessment of Dietary Intake

Dietary intake was assessed based on two 24HRs. Both were collected by trained interviewers on non-consecutive days, during all seasons of the year and days of the week. The first 24HR was collected in the household and the second was collected by telephone. These 24HRs were collected using, respectively, the Multiple-Pass Method and the Automated Multiple Pass Method, which have been described in detail by Guenther et al. and Blanton et al. [17,18]. The Nutrition Data System for Research (NDSR) software, 2014 version for the analyses of dietary intake data was used. This software was created at the University of Minnesota by the Nutrition Coordinating Center (Minneapolis, MN, USA) [19]. The NDSR software uses the American food composition database developed by the United States Department of Agriculture (USDA) [20]. Brazilian food compositions were compared with the USDA table and, if necessary, the BCAA value in food was corrected [21].

The prevalence of inadequate dietary intake of BCAA was determined using the estimated average requirement (EAR) cut-off point approach, a simplification of the probability method, which estimates the proportion of individuals with usual intakes below the EAR (median requirement).

Food contributors to BCAA intake were investigated using the method of considering the sampling design, proposed by Block et al. [22]. This method estimates the corresponding percentage of foods or food groups consumed by the population from the total intake of the nutrient assessed. A total of 45 groups were arranged by similarity in nutritional composition of BCAA.

The misreporting percentage was estimated using the equation: Energy intake—EER (estimated energy requirements)/EER∙100 [23]. The equations used to calculate EER on an individual basis were from Institute of Medicine of the National Academies [24].

### 2.4. Statistical Analyses

The characteristics of subjects were presented as medians and interquartile range (IQR) for continuous variables, and percentages for categorical variables. The total BCAA was defined as the sum of the energy-adjusted dietary Leu, Ile and Val. The usual BCAA intake was estimated using the Multiple Source Method (MSM) [25]. This web-based tool provides usual food intake distributions by linking the probability and the amount of consumption with the integration of covariates into the model [26]. Multiple linear regression models were used to evaluate the associations between the total and each individual’s BCAA intake with demographic, socioeconomic and lifestyle variables in all age groups (adolescents, adults and older adults). For these regression models, the total and each BCAA were adjusted for total energy intake using the nutrient residual model [27]. Furthermore, all models were adjusted for misreporting and the models were accepted after residual analysis. 

All analyses considered the complexity of the sample design and were performed with STATA version 13.0 (StataCorp LLC., College Station, TX, USA), considering *p*-values < 0.05 as statistically significant.

## 3. Results

The sample (*n* = 1662) was predominantly composed of the female sex (56.8%), the adults age group (35.2%), never smoked status (69.5%), self-reported white skin colour (58.0%) and non-consumer of alcohol beverages (58.3%). The median of the household head’s education was eight years (IQR 4–11) and household per capita income was US$275.0 (IQR 164.0–476.0). Excess body weight was observed in 35.7% of the individuals and 84.7% had insufficient leisure time physical activity.

**Figure 1:**
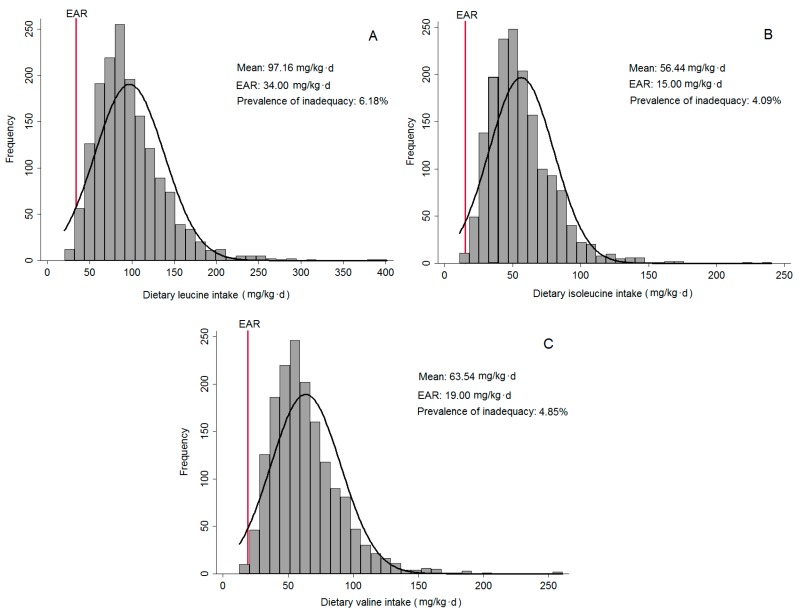
Distribution of dietary intake of leucine (**A**); isoleucine (**B**); and valine (**C**) of residents of the urban area of São Paulo. ISA-Capital, Sao Paulo, Brazil, 2008.

The mean of total BCAA intake was 217.14 mg/kg·day, with 97.16 mg/kg·day from Leu; 56.44 mg/kg·day from Ile; and 63.54 mg/kg·day from Val. In Figure 1, it is possible to observe that deficiency of BCAA is low in the residents of São Paulo. Regarding age groups, the mean of total protein was 85.64 g/day for adolescents, 80.01 g/day for adults and 69.81 g/day for older adults. Considering the total BCAA by groups, the means for adolescents, adults and older adults were, respectively, 268.04 mg/kg·day, 203.13 mg/kg·day and 178.37 mg/kg·day.

The association between individuals’ BCAA (Leu, Ile and Val) and the total BCAA with socioeconomic, demographic and lifestyle conditions are shown in Table 1, Table 2 and Table 3. After further adjustment for potential confounders, in adolescents, Leu, Ile, Val and total BCAA intakes were negatively associated with the female sex, non-white race and were positively associated with household per capita income. In adults, Leu, Ile, Val, and total BCAA intakes were negatively associated with the female sex and former smoker status and were positively associated with household per capita income. No associations were observed for older adults.

The main food contributors to Leu, Ile, Val, and total BCAA intakes were unprocessed red meat, unprocessed poultry, bread and toast, and beans and rice (Table 4, Table 5 and Table 6).

## 4. Discussion

In this study, we observed consistent associations of BCAA consumption, including Leu, Ile and Val, with socioeconomic, demographic and lifestyle factors in adolescents and adults. Compared to dietary reference intake (DRI), residents of São Paulo appear to have a low prevalence of inadequacy. It is important to emphasize that some authors have raised questions about the DRI for BCAA intake and suggested higher values of requirement. Riazi et al. [28], for example, indicated a total BCAA requirement for adults of 144 mg/kg·day, a value more than twice as high as the estimation by the DRI Committee (68 mg/kg·day). A group from the University of Toronto had suggested values 48% higher than the DRI value for young children [29]. Some factors could be responsible for the distinct amino acids requirement, such as energy level, protein (or nitrogen) level, and fibre type [30]. These facts impair the ability to determine precise requirements for amino acids in humans and animal models [31,32].

In adults, protein requirement estimations has been described in the literature and depend on one of two main approaches, namely, the factorial method and nitrogen balance. The BCAA-requirement estimation methods have limitations and are inconclusive. The DRI community uses the average of the values found by the methods [24]. Besides this, considering all values of BCAA intake observed here, it is important to investigate the tolerable upper intake level of these amino acids for each age, mainly in older adults, once BCAA consumption can be linked to NCD.

Regardless of age, women had a lower association with the consumption of these amino acids when compared with men. Micha et al. [33] observed a similar result when quantifying global intake of key foods related to NCD in adults from 187 countries and supposed that women consumed less unprocessed red meat (−4.2 g/day) in the Tropical Latin America region—unprocessed red meat is one of the main sources of BCAA. The lower protein consumption of women could be explained by the lower amount of food consumed in this sex group [34], probably because females generally need a lower energy intake than males, due to smaller average body weight and lower resting metabolic rate [35]. Despite these differences between the sexes, EAR does not consider different values for both sexes, which highlights this difference.

Former smoker status was associated with higher intake of individual and total BCAA in adults. Smoking cessation appears to be associated with being overweight and having a higher intake of energy (calories), cholesterol, saturated fatty acids, and alcohol [36]. In addition, being overweight or obese may contribute to a higher consumption of animal protein; western dietary habits; lower consumption of fruits, vegetables, whole grains; and a higher consumption of sweets [37,38].

The results obtained in the present study pointed to the association between self-reported race, household per capita income, and higher intake of all these amino acids. This association corroborates with the results of Carvalho et al. [39], which showed that Brazilians ingest more red meat for all age groups and these results are associated with higher household per capita income. The high consumption of red meat has been described in the literature. Souza et al. [40] observed during the National Dietary Survey, which used the database of the National Family Budgets Survey, that the percentage of red meat consumption varies from 43% to 50%. It is important to emphasize that meat, rice, beans, coffee and bread constitute the dietary pattern of the Brazilian population.

Our findings demonstrate that unprocessed red meat is the main source of dietary Leu, Ile, Val and total BCAA intake for all age groups, constituting 22% of food intake. Regarding the BCAA intake, our results were similar to the US adult population, in that the major food contributors were red meat (~37%), milk (~12%) and fish (~8%) [6]. However, these results are different from those observed in Japan, whose lowest food contributor with total BCAA intake in the adult population was red meat with approximately 14.9% of consumption in men and 13.7% in women [5].

In older adults, no significant results were found. This age group generally consumes less protein and some of the major dangers of this is the loss of muscle mass, strength and sarcopenia, which is a function that progressively occurs with aging. Several studies have identified animal protein, which contains essential amino acids, as a key nutrient for muscle health in older adults. These populations are less responsive to the anabolic stimulus of low doses of amino acid intake compared to younger individuals [41]. Therefore, the requirement for older adults needs to be higher for better anabolic responsiveness and the consumption of high-quality proteins must be proposed.

In a recent population-based cohort study, Zheng et al. [6] showed that high circulating levels of BCAA can be a biomarker of type 2 diabetes and a risk marker of other NCDs. In this context, over nutrition affects the rising of BCAA circulating in the plasma, leading to increase flux of these amino acids through their catabolic pathways [6]. In humans, these amino acids play regulatory roles in insulin and glucose metabolism. Thus, diet is an important source of these amino acids, and meat is an important food item for human nutrition; however, excessive meat intake, especially red and processed red meat, has been linked to the risk of cancer and to potentially carcinogenic substances [42].

Our study has limitations. First, this is a cross-sectional study, consequently, it is impossible to determine causality between the factors evaluated and BCAA intake. In addition, the dietary assessment used was the 24HR, which is a method that has a source of errors such as recollection of memory, omissions and possible errors in portion size estimation. However, we sought to minimize the influence of the potentially confounding variables. We measured the database from two 24HRs, through the Multiple Pass Method and finally, we used the MSM to estimate usual intake.

## 5. Conclusions

Individuals with a higher household per capita income, of the male sex, white race and those who were former smokers are the groups that are most vulnerable to higher Leu, Ile, Val, and total BCAA intake. These characteristics may be used to better target the planning of public health policies. 

## Figures and Tables

**Table 1:** Demographic, socioeconomic and lifestyle factors associated with branched chain amino acid intake in adolescents, ISA Capital, Sao Paulo, Brazil, 2008.

Variables	Leucine		Isoleucine		Valine		Total BCAA	
	β	95% CI	β	95% CI	β	95% CI	β	95% CI
*Sex (Ref. male)*								
Female	−0.43	−0.71; −0.16	−0.25	−0.42; −0.09	−0.28	−0.46; −0.11	−0.98	−1.59; −0.37
*Race (Ref. white)*								
No white	−0.24	−0.47; −0.02	−0.16	−0.29; −0.02	−0.17	−0.32; −0.03	−0.58	−1.09; −0.08
*Smoking status (Ref. nonsmoker)*								
Former smoker	−0.29	−0.91; 0.31	−0.15	−0.56; 0.24	−0.19	−0.58; 0.20	−0.64	−2.05; 0.76
Current smoker	−0.71	−1.50; 0.07	−0.44	−0.92; 0.04	−0.47	−1.00; 0.05	−1.62	−3.43; 0.17
*Alcohol consumption (Ref. no consumer)*								
Consumer	0.03	−0.20; 0.28	0.01	−0.13; 0.16	0.00	−0.14; 0.16	0.06	−0.49; 0.61
Household per capita income (US$ per month) *	0.02	0.01; 0.04	0.01	0.01; 0.02	0.01	0.01; 0.02	0.06	0.02; 0.10
*Household head education*	−0.00	−0.04; 0.03	−0.00	−0.02; 0.01	−0.00	−0.03; 0.01	−0.01	−0.01; 0.00
*Body Mass Index (Ref. without excess body weight)* ^‡^								
With excess body weight	−0.22	−0.47; 0.02	−0.10	−0.26; 0.04	−0.12	−0.29; 0.04	−0.45	−1.03; 0.11
*Leisure time physical activity (Ref. Insufficiently active)*								
Sufficiently active ^†^	−0.29	−0.61; 0.03	−0.16	−0.35; 0.01	−0.16	−0.38; 0.04	−0.63	−1.35; 0.08

Ref, reference. Branched chain amino acids (BCAA) intakes were adjusted according to the individual energy intake [27] and intra-individual variability [25]. All models were adjusted for misreporting. * The values of household per capita income were divided per 100 US$. ^‡^ BMI classifications: underweight, BMI < 3rd percentile; healthy weight, BMI > 3rd percentile and <85th percentile; overweight, BMI > 85th percentile and <97th percentile; obese, BMI > 97th percentile. ^†^ Sufficient physical activity: moderate-intensity exercise for at least 30 min daily on 5 day/week or vigorous-intensity exercise for at least 20 min daily on 3 day/week; otherwise, it was considered as insufficient physical activity.

**Table 2:** Demographic, socioeconomic and lifestyle factors associated with branched chain amino acid intake in adults, ISA Capital, São Paulo, Brazil, 2008.

Variables	Leucine		Isoleucine		Valine		Total BCAA	
	β	95% CI	β	95% CI	β	95% CI	β	95% CI
*Sex (Ref. male)*								
Female	−0.35	−0.61; −0.10	−0.21	−0.36; −0.06	−0.24	−0.40; −0.07	−0.81	−1.38; −0.24
*Race (Ref. white)*								
No white	−0.02	−0.26; 0.22	−0.02	−0.17; 0.12	−0.02	−0.18; 0.13	−0.06	−0.61; 0.48
*Smoking status (Ref. nonsmoker)*								
Former smoker	0.36	0.04; 0.67	0.24	0.04; 0.44	0.23	0.03; 0.43	0.83	0.12; 1.55
Current smoker	−0.11	−0.46; 0.23	−0.07	−0.29; 0.13	−0.09	−0.32; 0.13	−0.29	−1.08; 0.50
*Alcohol consumption (Ref. no consumer)*								
Consumer	0.05	−0.18; 0.28	0.03	−0.11; 0.17	0.02	−0.12; 0.17	0.10	−0.41; 0.63
Household per capita income (US$ per month) *	0.02	0.01; 0.04	0.01	0.01; 0.02	0.01	0.01; 0.03	0.64	0.02; 0.10
*Household head education*	−0.04	−0.08; 0.00	−0.02	−0.04; 0.00	−0.02	−0.05; 0.00	−0.09	−0.18; 0.00
*Body Mass Index (Ref. without excess body weight)* ^‡^								
With excess body weight	−0.12	−0.33; 0.09	−0.08	−0.21; 0.04	−0.07	−0.20; 0.06	−0.28	−0.76; 0.19
*Leisure time physical activity (Ref. Insufficiently active)*								
Sufficiently active ^†^	0.01	−0.28; 0.30	0.01	−0.28	−0.00	−0.30; 0.30	0.00	−1.06; 1.08

Ref, reference. BCAA intakes were adjusted according to the individual energy intake [27] and intra-individual variability [25]. All models were adjusted for misreporting. * The values of household per capita income were divided per 100 US$. ^‡^ BMI classifications: underweight, BMI < 18.5 kg/m^2^; healthy weight, BMI 18.5–24.9 kg/m^2^; overweight, BMI 25.0–29.9 kg/m^2^; obese, BMI ≥ 30 kg/m^2^. ^†^ Sufficient physical activity: moderate-intensity exercise for at least 30 min daily on 5 day/week or vigorous-intensity exercise for at least 20 min daily on 3 day/week; otherwise, it was considered as insufficient physical activity.

**Table 3:** Demographic, socioeconomic and lifestyle factors associated with branched chain amino acid intake in elderly adults, ISA Capital, São Paulo, Brazil, 2008.

Variables	Leucine		Isoleucine		Valine		Total BCAA	
	β	95% CI	β	95% CI	β	95% CI	β	95% CI
*Sex (Ref. male)*								
Female	−0.14	−0.31; 0.01	−0.06	−0.16; 0.03	−0.08	−0.19; 0.02	−0.30	−0.67; 0.06
*Race (Ref. white)*								
No white	0.13	−0.11; 0.38	0.06	−0.08; 0.20	0.07	−0.96; 0.23	0.26	−0.29; 0.83
*Smoking status (Ref. nonsmoker)*								
Former smoker	0.02	−0.19; 0.24	0.02	−0.10; 0.15	0.01	−0.12; 0.14	0.05	−0.43; 0.53
Current smoker	0.19	−0.16; 0.55	0.13	−0.09; 0.36	0.13	−0.10; 0.36	0.46	−0.35; 1.28
*Alcohol consumption (Ref. no consumer)*								
Consumer	0.07	−0.15; 0.31	0.02	−0.10; 0.16	0.03	−0.11; 0.18	0.14	−0.37; 0.66
Household per capita income (US$ per month) *	0.00	−0.02; 0.02	0.00	−0.01; 0.01	0.00	−0.01; 0.01	0.00	−0.05; 0.06
*Household head education*	−0.00	−0.04; 0.02	−0.00	−0.02; 0.01	−0.00	−0.02; 0.01	0.01	0.00; 0.03
*Body Mass Index (Ref. without excess body weight)* ^‡^								
With excess body weight	0.02	−0.22; 0.27	0.00	−0.13; 0.14	0.01	−0.14; 0.16	0.03	−0.50; 0.58
*Leisure time physical activity (Ref. Insufficiently active)*								
Sufficiently active ^†^	0.19	−0.05; 0.43	0.13	−0.02; 0.29	0.14	−0.02; 0.31	0.46	−0.10; 1.04

Ref, reference. BCAA intakes were adjusted according to the individual energy intake [27] and intra-individual variability [25]. All models were adjusted for misreporting. * The values of household per capita income were divided per 100 US$. ^‡^ BMI classifications: underweight, BMI < 23 kg/m^2^; healthy weight, BMI 23.0–27.9 kg/m^2^; overweight, BMI 28–29.9 kg/m^2^; obese, BMI ≥ 30 kg/m^2^. ^†^ Sufficient physical activity: moderate-intensity exercise for at least 30 min daily on 5 day/week or vigorous-intensity exercise for at least 20 min daily on 3 day/week; otherwise, it was considered as insufficient physical activity.

**Table 4:** Main food contributors to dietary intake of branched chain amino acids in adolescents, ISA Capital, São Paulo, Brazil, 2008.

Rank	Leucine			Isoleucine			Valine			Total BCAA		
	Food	Median (g)	%	Food	Median (g)	%	Food	Median (g)	%	Food	Median (g)	%
1	Unprocessed red meat	105.0	22.4	Unprocessed red meat	105.0	22.4	Unprocessed red meat	105.0	21.1	Unprocessed red meat	105.0	22.0
2	Unprocessed poultry	80.0	9.0	Unprocessed poultry	80.0	10.8	Unprocessed poultry	80.0	9.2	Unprocessed poultry	80.0	9.5
3	Savoury baked	113.1	7.8	Savoury baked	113.1	7.4	Savoury baked	113.1	7.5	Savoury baked	113.1	7.6
4	Bread and toast	50.0	6.7	Bread and toast	50.0	6.3	Bread and toast	50.0	6.4	Bread and toast	50.0	6.5
5	Beans	43.0	6.0	Beans	43.0	5.7	Rice	150.0	6.1	Beans	43.0	5.9
6	Rice	150.0	5.4	Whole milk	180.4	5.4	Beans	43.0	6.0	Rice	150.0	5.5
7	Whole milk	180.4	5.0	Rice	150.0	4.9	Whole milk	180.4	5.6	Whole milk	180.4	5.3
8	Processed red meat	56.0	4.2	Processed pork	47.5	4.2	Processed red meat	56.0	4.0	Processed red meat	56.0	4.1
9	Processed pork	47.5	3.9	Processed red meat	56.0	4.1	Processed pork	47.5	4.0	Processed pork	47.5	4.0
10	Unprocessed pork	100.0	3.2	Unprocessed pork	100.0	3.2	Unprocessed pork	100.0	3.3	Unprocessed pork	100.0	3.2
11	Sandwiches	124.3	3.1	Sandwiches	124.3	3.0	Sandwiches	124.3	3.0	Sandwiches	124.3	3.0
12	Yellow cheese	30.0	2.4	Yellow cheese	30.0	2.5	Yellow cheese	30.0	2.8	Yellow cheese	30.0	2.5
13	Fresh pasta	220.0	2.2	Candies	50.0	2.2	Candies	50.0	2.3	Candies	50.0	2.2
14	Unprocessed fish	120.0	2.2	Unprocessed fish	120.0	2.2	Unprocessed fish	120.0	2.1	Unprocessed fish	120.0	2.2
15	Candies	50.0	2.2	Fried snacks	60.0	2.1	Fresh pasta	220.0	2.1	Fresh pasta	220.0	2.1
% total			85.7			86.4			85.5			85.6

**Table 5:** Main food contributors to dietary intake of branched chain amino acids in adults, ISA Capital, São Paulo, Brazil, 2008.

Rank	Leucine			Isoleucine			Valine			Total BCAA		
	Food	Median (g)	%	Food	Median (g)	%	Food	Median (g)	%	Food	Median (g)	%
1	Unprocessed red meat	100.0	22.4	Unprocessed red meat	100.0	22.1	Unprocessed red meat	100.0	21.2	Unprocessed red meat	100.0	22.0
2	Unprocessed poultry	80.0	14.8	Unprocessed poultry	80.0	17.5	Unprocessed poultry	80.0	15.0	Unprocessed poultry	80.0	15.5
3	Bread and toast	50.0	6.3	Beans	50.0	5.9	Rice	124.0	7.0	Rice	124.0	6.3
4	Beans	43.0	6.3	Bread and toast	43.0	5.9	Beans	43.0	6.4	Beans	43.0	6.2
5	Rice	124.0	6.3	Rice	124.0	5.6	Bread and toast	50.0	6.0	Bread and toast	50.0	6.1
6	Unprocessed fish	162.5	5.0	Unprocessed fish	162.5	4.9	Unprocessed fish	162.5	4.9	Unprocessed fish	162.5	4.9
7	Savoury baked	100.0	4.7	Savoury baked	100.0	4.4	Savoury baked	100.0	4.5	Savoury baked	100.0	4.5
8	Whole milk	123.7	3.9	Whole milk	123.7	4.2	Whole milk	123.7	4.4	Whole milk	123.7	4.1
9	Processed pork	30.0	3.7	Processed pork	30.0	3.7	Processed pork	30.0	3.6	Processed pork	30.0	3.6
10	Unprocessed pork	90.0	2.6	Unprocessed pork	90.0	2.6	Unprocessed pork	90.0	2.7	Unprocessed pork	90.0	2.6
11	Yellow cheese	30.0	2.4	Yellow cheese	30.0	2.3	Yellow cheese	30.0	2.6	Yellow cheese	30.0	2.4
12	Processed red meat	52.0	2.2	Processed red meat	52.0	2.2	Processed red meat	52.0	2.1	Processed red meat	52.0	2.2
13	Stuffed pasta	190.0	2.2	Stuffed pasta	190.0	2.0	Stuffed pasta	190.0	2.1	Stuffed pasta	190.0	2.1
14	Fresh pasta	190.0	1.7	Eggs	190.0	1.6	Candies	50.0	1.6	Fresh pasta	190.0	1.6
15	Sandwiches	82.0	1.6	Candies	82.0	1.6	Eggs	50.0	1.6	Candies	50.0	1.6
% total			86.1			86.5			85.7			85.7

**Table 6:** Main food contributors to dietary intake of branched chain amino acids in older adults, ISA Capital, São Paulo, Brazil, 2008.

Rank	Leucine			Isoleucine			Valine			Total BCAA		
	Food	Median (g)	%	Food	Median (g)	%	Food	Median (g)	%	Food	Median (g)	%
1	Unprocessed red meat	100.0	20.7	Unprocessed red meat	100.0	20.5	Unprocessed red meat	100.0	19.5	Unprocessed red meat	100.0	20.3
2	Unprocessed poultry	86.0	13.5	Unprocessed poultry	80.0	16.1	Unprocessed poultry	65.0	13.7	Unprocessed poultry	65.0	14.2
3	Bread and toast	20.0	7.4	Beans	43.0	6.9	Rice	116.2	7.6	Bread and toast	50.0	7.2
4	Rice	45.0	6.8	Bread and toast	50.0	6.6	Bread and toast	50.0	7.0	Rice	116.2	6.8
5	Beans	91.3	6.5	Rice	124.0	6.2	Whole milk	112.9	6.9	Whole milk	112.9	6.5
6	Whole milk	60.0	6.2	Unprocessed fish	162.5	6.1	Beans	43.0	6.5	Beans	43.0	6.4
7	Processed red meat	150.0	3.5	Savoury baked	100.0	3.4	Processed pork	30.0	3.3	Processed red meat	50.0	3.4
8	Processed pork	25.0	3.4	whole milk	123.7	3.4	Processed red meat	50.0	3.3	Processed pork	30.0	3.4
9	Skimmed milk	77.3	3.1	Processed pork	30.0	2.8	Unprocessed pork	75.0	2.9	Skimmed milk	133.3	2.9
10	White cheese	16.8	2.9	Unprocessed pork	90.0	2.7	Skimmed milk	133.3	2.8	Unprocessed pork	75.0	2.8
11	Unprocessed pork	15.0	2.8	Yellow cheese	30.0	2.6	White cheese	30.0	2.6	White cheese	30.0	2.7
12	Yellow cheese	10.8	2.2	Processed red meat	52.0	2.2	Yellow cheese	20.0	2.4	Yellow cheese	20.0	2.2
13	Soups	30.0	2.1	Stuffed pasta	190.0	2.1	Soups	325.0	2.1	Soups	325.0	2.2
14	Savoury baked	50.0	2.0	Eggs	50.0	2.0	Unprocessed fish	106.1	1.9	Unprocessed fish	106.1	2.0
15	Unprocessed fish	1.7	2.0	Candies	50.0	1.9	Savoury baked	90.1	1.9	Savoury baked	90.1	2.0
% total			85.1			85.5			84.4			85.0
